# Extensive Pelvic Venous Malformation in a Child With Recurrent Hematochezia

**DOI:** 10.1002/ccr3.72271

**Published:** 2026-03-17

**Authors:** Markus Denzinger, Patricia Reis Wolfertstetter, Pirmin Zöhrer, Christian Knorr

**Affiliations:** ^1^ Faculty of Medicine University of Regensburg Regensburg Germany; ^2^ University Children's Hospital Regensburg (KUNO) Regensburg Germany; ^3^ Children's Hospital of Eastern Switzerland St. Gallen Switzerland

**Keywords:** cutaneous vascular malformation, gastrointestinal bleeding, gastrointestinal vascular malformation, hematochezia, vascular malformation

## Abstract

The coexistence of unexplained rectal bleeding and a cutaneous vascular malformation should raise suspicion of an underlying combined vascular malformation. Awareness of this association and a multidisciplinary diagnostic approach are essential to avoid misdiagnosis and ensure appropriate patient management.

A 3‐year‐old boy presented with a 6‐month history of recurrent, painless hematochezia. Initially, the bleeding episodes were infrequent but increased in frequency over the following months. During this period, his parents also observed a 1.5 cm soft, bluish‐red perianal lesion. In the weeks preceding admission, a large cutaneous lesion (initially clinically interpreted as a hemangioma) on the left thigh began to bleed intermittently as well. Apart from this lesion, no external abnormalities or malformations were observed.

Laboratory testing at admission revealed severe anemia (hemoglobin 5.8 g/dL). The patient had previously received a blood transfusion at an external hospital, where an abdominal ultrasound had shown no visceral vascular abnormalities. Esophagogastroduodenoscopy showed no abnormalities. However, colonoscopy revealed nonspecific mucosal changes consistent with indeterminate colitis, prompting the initiation of mesalazine therapy.

Despite treatment, the patient continued to pass bloody stools during hospitalization. Endoscopic biopsies showed no diagnostic pathology. An exploratory laparoscopy was performed to rule out Meckel's diverticulum. Intraoperatively, a diffuse vascular malformation infiltrating the rectum and sigmoid colon was identified (Figure 1A,B). Postoperative MRI confirmed an extensive venous vascular malformation extending from the cutaneous anomaly on the thigh through the obturator foramen into the pelvis and involving the bowel wall (Figure 1C).

**FIGURE 1:**
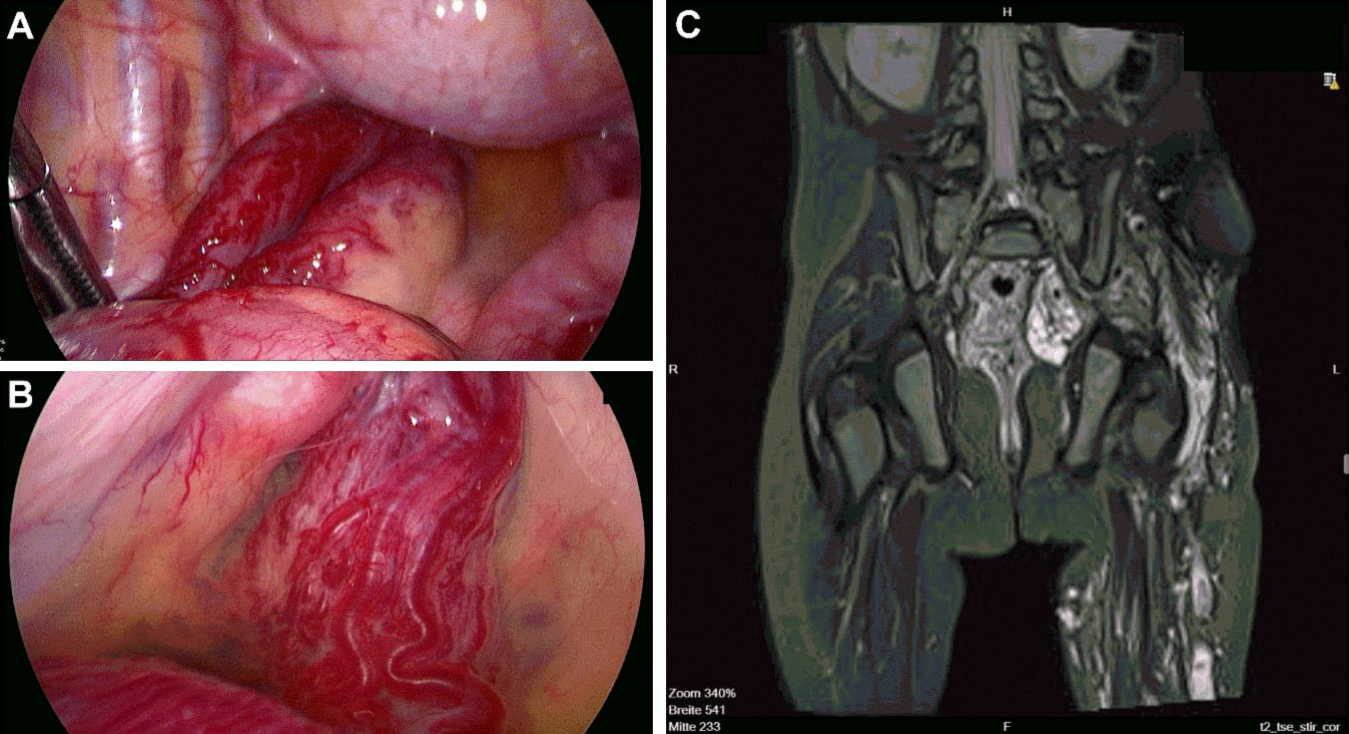
(A) Intraoperative view showing a venous vascular malformation infiltrating the sigmoid colon (visible in the central part of the image). (B) Intraoperative detail of the rectal involvement with diffuse subserosal vascular channels. (C) Coronar T2‐weighted MRI demonstrating the extensive venous malformation extending from the cutaneous capillary malformation on the left thigh through the obturator foramen into the pelvis, involving the rectal and sigmoid wall.

Vascular malformations of the gastrointestinal tract are rare and frequently misdiagnosed due to their deep location and variable presentation [1, 2, 3]. In this case, the coexistence of a cutaneous vascular malformation with gastrointestinal bleeding raised suspicion of a syndromic vascular anomaly. Cross‐sectional imaging was critical for defining the extent and character of the lesion. Importantly, histopathology may be nonspecific or even normal, particularly when mucosal involvement is minimal and the malformation is predominantly submucosal or transmural.

This case highlights the importance of considering vascular malformations in the differential diagnosis of recurrent lower gastrointestinal bleeding in children, especially when associated with cutaneous findings or unexplained anemia. A multidisciplinary approach, including pediatric surgery, radiology, and gastroenterology, is essential for accurate diagnosis and treatment planning.

## Author Contributions

**Markus Denzinger:** conceptualization, data curation, investigation, project administration, supervision, writing – original draft, writing – review and editing. **Patricia Reis Wolfertstetter:** writing – original draft, writing – review and editing. **Pirmin Zöhrer:** writing – original draft, writing – review and editing. **Christian Knorr:** project administration, writing – original draft, writing – review and editing.

## Funding

The authors have nothing to report.

## Consent

The patient's written informed consent was obtained prior to publication.

## Conflicts of Interest

The authors declare no conflicts of interest.

## Data Availability

The data supporting the findings of this case are available from the corresponding author upon reasonable request.
